# The General Solution of Singular Fractional-Order Linear Time-Invariant Continuous Systems with Regular Pencils

**DOI:** 10.3390/e20060400

**Published:** 2018-05-23

**Authors:** Iqbal M. Batiha, Reyad El-Khazali, Ahmed AlSaedi, Shaher Momani

**Affiliations:** 1 Department of Mathematics, Faculty of Science, University of Jordan, Amman 11942, Jordan; 2 ECCE Department, Khalifa University, Abu-Dhabi 127788, United Arab Emirates; 3 Department of Nonlinear Analysis and Applied Mathematics (NAAM) Research Group, Faculty of Science, King Abdulaziz University (KAU), Jeddah 21589, Saudi Arabia

**Keywords:** fractional calculus, Adomian decomposition, Mittag–Leffler function, descriptor fractional linear systems, regular pencils, Schur factorization

## Abstract

This paper introduces a general solution of singular fractional-order linear-time invariant (FoLTI) continuous systems using the Adomian Decomposition Method (ADM) based on the Caputo's definition of the fractional-order derivative. The complexity of their entropy lies in defining the complete solution of such systems, which depends on introducing a method of decomposing their dynamic states from their static states. The solution is formulated by converting the singular system of regular pencils into a recursive form using the sequence of transformations, which separates the dynamic variables from the algebraic variables. The main idea of this work is demonstrated via numerical examples.

## 1. Introduction

A dynamical system represented by differential equations with non-integer order derivatives is denoted as a fractional-order system. In general, most practical systems are best described by fractional-order dynamics (FoD), where the integer-order representation of such systems is considered as a special case. Recently, different types of problems of fractional-order dynamical systems have been considered in the literature [1,2]. Time-domain system identification using the fractional-order models was initiated in the late nineties. Several methods of discretizing the fractional-order differential equation using Grunwald–Letnikov (GL) approximation or phase assignment technique can be found in [3,4], while another biquadratic approximation of the fractional-order Laplacian operator based on the flatness of the phase frequency response at its center frequency is discussed in [5]. Furthermore, the state–space representations of fractional-order systems have been broadly used to investigate system stability, observability and controllability [6,7,8]. The generalization of FoD have allowed it to flourish in many fields of applications, such as control theory, communication systems and applied mathematics [9,10]. 

The singular (descriptor) fractional-order system of differential equations plays an important role in many applications, such as electric networks, economics, optimization problems, analysis of control systems, constrained mechanics, aircraft and robot dynamics, biology and large-scale systems [9,10]. Many continuous or discrete-time systems are usually described by complete dynamical states that vary with time, which have wide applications in social sciences, chaotic systems, economics, electrical networks, information theory and medical sciences [11,12,13,14,15,16]. Since the singular systems enjoy static and dynamic states, the complexity of their entropy depends on the methods of decomposing these states from each other to completely identify the analytical solution of such systems.

The solution of singular systems with regular and singular pencils was discussed in references [17,18,19,20,21], while the optimal solution of a class of singular linear systems of regular and singular pencils that have non-consistent linear systems of nabla difference equations with non-consistent initial conditions was discussed in reference [22]. The relationship between the solutions of an initial value problem of a linear singular system of fractional nabla difference equations, its proper dual system and its transposed dual system as well as introduced necessary and sufficient conditions for the existence and uniqueness of their solution were thoroughly investigated in reference [23]. The initial value problem of a class of non-homogeneous singular systems of fractional nabla difference equations with constant matrix coefficients was investigated in reference [24], which considered two cases: square coefficient matrices with a singular leading coefficient and regular pencils; and square and non-square matrices of singular pencils. 

In this work, we only considered singular linear systems with regular pencils. The case of the systems of singular pencils is left for further development. To find the general solution of fractional-order singular systems, the Adomian Decomposition Method (ADM) [22,23] is extended by first introducing the general solution of the regular commensurate fractional-order linear-time invariant (FoLTI) continuous systems, which is described by the following general form:

(1a) $$D^{\alpha }x\left(t\right)=Ax\left(t\right)+Bu\left(t\right),0<\alpha \leq 1,$$

(1b) y(t)=Cx(t)+Du(t),

where $x\left(t\right)\in {\mathbb{R}}^{n},\;u\left(t\right)\in {\mathbb{R}}^{m}\;\mathrm{and}\;y\left(t\right)\in {\mathbb{R}}^{p}$ are the system states, the input, and output vectors, respectively; while $A\in {\mathbb{R}}^{n\times n},\;B\in {\mathbb{R}}^{n\times m},\;C\in {\mathbb{R}}^{p\times n}\;\mathrm{and}\;D\in {\mathbb{R}}^{p\times m}$ are the system constant matrices.

The ADM is extended here to obtain the solution of a singular FoLTI continuous system that has the following general form:

(2a) $$ED^{\alpha }x\left(t\right)=Ax\left(t\right)+Bu\left(t\right),0<\alpha \leq 1,$$

(2b) y(t)=Cx(t)+Du(t),

where $E\in {\mathbb{R}}^{n\times n}$ is a singular matrix; $x\left(t\right)\in {\mathbb{R}}^{n}$ is the pseudo-state; $u\left(t\right)\in {\mathbb{R}}^{m}$ is the control input; $y\left(t\right)\in {\mathbb{R}}^{p}$ is the output; and $A\in {\mathbb{R}}^{n\times n},\;B\in {\mathbb{R}}^{n\times m}\;\mathrm{and}\;C\in {\mathbb{R}}^{p\times n}$ with rank C=p.

**Definition** **1.** [24] *The matrix pencil* sE−A*, where E and A* $\in {\mathbb{R}}^{n\times q}$ *and for an arbitrary* s∈ℂ*, is called:*

*(1)*  *Regular when*n=q*and det*(sE−A)≠0,
*(2)*  *Singular when*n=q*or*n≠q*and det*(sE−A)=0.

In this work, we considered the class of regular pencils with a singular matrix *E*. Singular fractional-order systems consist of coupled differential and algebraic equations. The control of singular fractional-order systems is not well-flourished compared to that of the conventional dynamical systems. However, it is possible to use the sequence of transformations to decouple the differential and the algebraic parts of the system from each other, thus enabling the application of the standard state–space control theory to a dynamical subsystem of a lower order [24,25,26,27].

There are three main steps used to decouple the system’s static and dynamic parts from each other. The first one involves using the generalized Schur decomposition method, the second one involves solving a coupled Sylvester equation and the third one involves constructing well-defined transformation matrices [28,29]. The first step is thoroughly investigated using numerical linear algebra. Various existing methods for transforming a matrix into a Jordan-Schur form and a matrix pencil into a Weierstrass–Schur form have been investigated in reference [28]. These methods are extended to extract the partial information that corresponds to the dominant eigenvalues from large-scale matrices and matrix pencils. The solution and perturbation analysis of a coupled Sylvester equation is presented in reference [29]. The Schur method and the Hessenberg–Schur method are extended for a coupled Sylvester equation, which is transformed into a standard Sylvester equation. This equation is solved using standard techniques presented in [28,29].

This work is outlined as follows. In the next section, the necessary definitions and preliminaries are introduced. Section 3 describes the ADM method. Section 4 introduces the solution of FoLTI systems with regular pencils. A recursive method to decompose the singular systems is introduced in Section 5, followed by numerical examples in Section 6. The summaries and concluding remarks are presented in Section 7.

## 2. Basic Definitions and Preliminaries

The Caputo definition of fractional-order derivatives is adopted in this work. It is a modification of the Riemann–Liouville definition and it has the advantage of only using the initial conditions that corresponds to integer-order derivatives, which is suitable for most physical systems [30,31,32]. The following definitions and preliminaries of fractional-order calculus are presented here for completeness.

**Definition** **2** **[9,10].***Let*f(t)*be an integrable piecewise continuous function on any finite subinterval of*(0,+∞). *Thus, the fractional integral of*f(t)*of order*α is defined as:

(3) $$J^{\alpha }f\left(t\right):=\frac{t^{\alpha -1}}{\Gamma \left(\alpha \right)}\times f\left(t\right)=\frac{1}{\Gamma \left(\alpha \right)}\int_{0}^{t}{\left(t-\tau \right)}^{\alpha -1}f\left(\tau \right)d\tau ,\;t>0,\;\alpha >0.$$

In this paper, we will use the following equality [20]:

(4) $$J^{\alpha }t^{\mu }=\frac{\Gamma \left(\mu +1\right)}{\Gamma \left(\mu +\alpha +1\right)}t^{\mu +\alpha },\;\alpha >0,\;\mu >-1,t>0.$$

**Definition** **3** **[9,10].***The Caputo fractional-order derivative is defined as:*

(5) $$D^{\alpha }f\left(t\right)=\frac{1}{\Gamma \left(M-\alpha \right)}\int_{0}^{t}\frac{f^{M}\left(\tau \right)}{{\left(t-\tau \right)}^{\alpha +1-M}}d\tau ,\;f^{M}\left(\tau \right)=\frac{d^{M}f\left(\tau \right)}{d\tau^{M}},$$

*where*Γ(·)*is the Gamma function and*M−1≤α<M, M∈ℕ.

**Definition** **4** **[8].***The Mittag–Leffler function of two parameters is defined by*:

(6) $$E_{\alpha ,\beta }\left(t\right)=\sum_{k=0}^{\infty }\frac{t^{k}}{\Gamma \left(k\alpha +\beta \right)}.$$

**Definition** **5** **[9].***The Mittag–Leffler matrix function of two parameters is defined by:*

(7) $$E_{\alpha ,\beta }\left(At^{\alpha }\right)=\sum_{k=0}^{\infty }\frac{A^{k}t^{\alpha k}}{\Gamma \left(k\alpha +\beta \right)},$$

*where*$A\in {\mathbb{R}}^{n\times n}$.

**Definition** **6** **[31].** *The system given by (1) or the pair* (E,A) *is said to be regular pencil if there exists a unique solution* x(t) *for a given initial condition.*

**Lemma** **1** **[32].***The system described by (1) or the pair*(E,A)*is said to be a regular pencil if and only if*$det\left(Es^{\alpha }-A\right)\neq 0$*, for*s∈ℂ.

## 3. Solution of FoLTI Systems Using ADM Method

In this section, we used the ADM method to obtain the general solution of fractional-order state equations of linear time-invariant continuous systems. See [25,26,27] for an overview of the ADM technique. In the subsequent discussion, consider the linear system described by (1a) and assume the definition of Caputo fractional-order derivative; i.e.,

(8) $$D^{\alpha }x\left(t\right)=Ax\left(t\right)+Bu\left(t\right),\;0<\alpha \leq 1,$$

with the initial condition:

(9) x(0)=v.

Notice that applying $J^{\alpha }$ (i.e., fractional-order integration of order α) on both sides of system (8) yields:

(10) $$x\left(t\right)=x\left(0\right)+AJ^{\alpha }x\left(t\right)+BJ^{\alpha }u\left(t\right).$$

To use the Adomian decomposition method, we assume that the general solution of (8) takes the general form of $x\left(t\right)=\sum_{k=0}^{\infty }x_{k}\left(t\right)$, in which:

(11) $$x_{0}\left(t\right)=v+BJ^{\alpha }u\left(t\right)$$

and

(12) $$x_{k}\left(t\right)=AJ^{\alpha }x_{k-1}\left(t\right),\;k\geq 1.$$

Now, from (11) and (12), one can obtain the following recursive formula for the system states:

(13) $$x_{1}\left(t\right)=J^{\alpha }\left[Av+ABJ^{\alpha }u\left(t\right)\right],\;x_{2}\left(t\right)=J^{2\alpha }\left[A^{2}v+A^{2}BJ^{\alpha }u\left(t\right)\right],\;\ldots ,\;x_{k}\left(t\right)=J^{k\alpha }\left[A^{k}v+A^{k}BJ^{\alpha }u\left(t\right)\right].$$

Therefore,

(14) $$x_{1}\left(t\right)=\frac{Av}{\Gamma \left(\alpha +1\right)}t^{\alpha }+ABJ^{2\alpha }u\left(t\right),\;x_{2}\left(t\right)=\frac{A^{2}v}{\Gamma \left(2\alpha +1\right)}t^{2\alpha }+A^{2}BJ^{3\alpha }u\left(t\right),\;\ldots ,\;x_{k}\left(t\right)=\frac{A^{k}v}{\Gamma \left(k\alpha +1\right)}t^{k\alpha }+A^{k}BJ^{\left(k+1\right)\alpha }u\left(t\right).$$

Since the general solution $x\left(t\right)=\sum_{k=0}^{\infty }x_{k}\left(t\right),$ Equation (14) then yields:

(15) $$x\left(t\right)=\sum_{k=0}^{\infty }\frac{{\left(At^{\alpha }\right)}^{k}}{\Gamma \left(k\alpha +1\right)}v+\;\sum_{k=0}^{\infty }A^{k}BJ^{\left(k+1\right)\alpha }u\left(t\right).$$

That is,

(16) $$x\left(t\right)=\sum_{k=0}^{\infty }\frac{{\left(At^{\alpha }\right)}^{k}}{\Gamma \left(k\alpha +1\right)}v+\;\sum_{k=0}^{\infty }A^{k}B\frac{1}{\Gamma \left(\left(k+1\right)\alpha \right)}\int_{0}^{t}{\left(t-\tau \right)}^{\left(k+1\right)\alpha -1}u\left(\tau \right)d\tau .$$

or,

(17) $$x\left(t\right)=\sum_{k=0}^{\infty }\frac{{\left(At^{\alpha }\right)}^{k}}{\Gamma \left(k\alpha +1\right)}v+\;\int_{0}^{t}\sum_{k=0}^{\infty }\frac{A^{k}{\left(t-\tau \right)}^{\left(k+1\right)\alpha -1}}{\Gamma \left(\left(k+1\right)\alpha \right)}Bu\left(\tau \right)d\tau .$$

Alternately, in terms of the Mittag–Leffler matrix functions of (7), one may rewrite (17) as follows:

(18) $$x\left(t\right)=E_{\alpha ,1}\left(At^{\alpha }\right)v+\left[t^{\alpha -1}E_{\alpha ,\alpha }\left(At^{\alpha }\right)\right]\;\left[Bu\left(\tau \right)\right].$$

Consequently, the general solution of system states, described by (8), can be written in the following general form:

(19) $$x\left(t\right)=\phi_{\alpha 0}\left(t\right)v+\int_{0}^{t}\phi_{\alpha }\left(t-\tau \right)Bu\left(\tau \right)d\tau ,\;x\left(0\right)=v$$

where

(20) $$\phi_{\alpha 0}\left(t\right)=E_{\alpha ,1}\left(At^{\alpha }\right)=\overset{\infty }{\underset{k=0}{\sum }}\frac{{\left(At^{\alpha }\right)}^{k}}{\Gamma \left(k\alpha +1\right)},$$

(21) $$\phi_{\alpha }\left(t\right)=t^{\alpha -1}E_{\alpha ,\alpha }\left(At^{\alpha }\right)=\sum_{k=0}^{\infty }\frac{A^{k}t^{\left(k+1\right)\alpha -1}}{\Gamma \left(\left(k+1\right)\alpha \right)}.$$

and the general solution of the system output is given by:

(22) $$y\left(t\right)=C\left\{E_{\alpha ,1}\left(At^{\alpha }\right)v+\left[t^{\alpha -1}E_{\alpha ,\alpha }\left(At^{\alpha }\right)\right]\;\left[Bu\left(t\right)\right]\right\}+Du\left(t\right).$$

## 4. The General Solution of FoLTI Singular Systems with Regular Pencils

The general solution of FoLTI singular continuous systems is usually obtained by first transforming the system into the canonical form [33,34,35], which enables one to easily decompose the static terms from the dynamic ones. The following lemmas are presented for completeness to derive the general solution of the FoLTI singular systems with regular pencils. To simplify the process of obtaining the general solution, the system matrices (E,A) with regular pencils may be both transformed into a triangular form with the zero eigenvalues of E placed at the lower right block.

**Lemma** **2** **[33].***Consider the following FoLTI singular continuous system:*

(23) $$ED^{\alpha }x\left(t\right)=Ax\left(t\right)+Bu\left(t\right),0<\alpha \leq 1,$$

*where*$E\in {\mathbb{R}}^{n\times n}$*is a singular matrix of rank*$n_{1}<n$, $x\left(t\right)\in {\mathbb{R}}^{n}$, $u\left(t\right)\in {\mathbb{R}}^{m}$, *and*$A\in {\mathbb{R}}^{n\times n},\;B\in {\mathbb{R}}^{n\times m}.$.

If (23) is regular, there exist non-singular matrices $P_{1},\;Q_{1}\in {\mathbb{R}}^{n\times n}$, such that:

(24) $$P_{1}EQ_{1}=\left[\begin{array}{cc} E_{1} & E_{2}\\ 0 & E_{3} \end{array}\right],\;P_{1}AQ_{1}=\left[\begin{array}{cc} J_{1} & J_{2}\\ 0 & J_{3} \end{array}\right],$$

where $E_{1}\in {\mathbb{R}}^{n_{1}\times n_{1}}$ is non-singular; $E_{3}\in {\mathbb{R}}^{n_{2}\times n_{2}}$ is an upper triangular matrix with all diagonal elements being zero; $J_{1}\in {\mathbb{R}}^{n_{1}\times n_{1}}$; $J_{3}\in {\mathbb{R}}^{n_{2}\times n_{2}}$ is non-singular and upper triangular; and $E_{2},\;J_{2}\in {\mathbb{R}}^{n_{1}\times n_{2}}$.

The generalized Schur decomposition given by (24) and the subsequent reordering of the diagonal elements of E may be conducted using “*qz*” MATLAB function to construct $E_{1}\;\mathrm{and}\;J_{1}$ as upper triangular matrices [32].

**Lemma** **3** **[36].***Consider (24), then there exist matrices*$L,\;R\in {\mathbb{R}}^{n_{1}\times n_{2}}$, *such that:*

(25) $$\left[\begin{array}{cc} I & L\\ 0 & I \end{array}\right]\left[\begin{array}{cc} E_{1} & E_{2}\\ 0 & E_{3} \end{array}\right]\left[\begin{array}{cc} I & R\\ 0 & I \end{array}\right]=\left[\begin{array}{cc} E_{1} & 0\\ 0 & E_{3} \end{array}\right]$$

*and*

(26) $$\left[\begin{array}{cc} I & L\\ 0 & I \end{array}\right]\left[\begin{array}{cc} J_{1} & J_{2}\\ 0 & J_{3} \end{array}\right]\left[\begin{array}{cc} I & R\\ 0 & I \end{array}\right]=\left[\begin{array}{cc} J_{1} & 0\\ 0 & J_{3} \end{array}\right].$$

**Lemma** **4** **[36].***Consider system (23), if this system is regular, then there exist non-singular matrices*$P,\;Q\in {\mathbb{R}}^{n\times n}$*such that the transformation:*

(27) $$PEQQ^{-1}D^{\alpha }x\left(t\right)=PAQQ^{-1}x\left(t\right)+PBu\left(t\right)$$

*yields the following structure:*

(28) $$\left[\begin{array}{cc} I_{n_{1}} & 0\\ 0 & N \end{array}\right]Q^{-1}D^{\alpha }x\left(t\right)=\left[\begin{array}{cc} F & 0\\ 0 & I_{n_{2}} \end{array}\right]Q^{-1}x\left(t\right)+\left[\begin{array}{c} H\\ K \end{array}\right]u\left(t\right),$$

*where*$N\in {\mathbb{R}}^{n_{2}\times n_{2}}$*is a nilpotent matrix,*$F\in {\mathbb{R}}^{n_{1}\times n_{1}}$*; and*$n_{1}$*is equal to the degree of the polynomial*det (Es−A),*such that*$n_{1}+n_{2}=n$*, and where:*

(29) $$PEQ=\left[\begin{array}{cc} I_{n_{1}} & 0\\ 0 & N \end{array}\right],\;PAQ=\left[\begin{array}{cc} F & 0\\ 0 & I_{n_{2}} \end{array}\right],\;PB=\left[\begin{array}{c} H\\ K \end{array}\right],$$

*where*$H\in {\mathbb{R}}^{n_{1}\times m}$, $K\in {\mathbb{R}}^{n_{2}\times m}$*and*s∈ℂ.

**Proof.**  According to Lemma 1, let $P_{1}\;\mathrm{and}\;Q_{1}$ be matrices such that $P=P_{3}P_{2}P_{1}$ and $Q=Q_{1}Q_{2}$, where:

(30) $$P_{2}\equiv \left[\begin{array}{cc} I & L\\ 0 & I \end{array}\right],\;P_{3}\equiv \left[\begin{array}{cc} E_{1}^{-1} & R\\ 0 & J_{3}^{-1} \end{array}\right],\;\mathrm{and}\;Q_{2}\equiv \left[\begin{array}{cc} I & R\\ 0 & I \end{array}\right],$$

where $P_{2},P_{3},\;Q_{2}\in {\mathbb{R}}^{n\times n}$. □

Now from Lemma 2, the matrices L and R satisfy:

(31) $$PEQ=\left[\begin{array}{cc} I & L\\ 0 & J_{3}^{-1}E_{3} \end{array}\right],\;PAQ=\left[\begin{array}{cc} E_{1}^{-1}J_{1} & R\\ 0 & I \end{array}\right],$$

where $N=J_{3}^{-1}E_{3}$ is a nilpotent matrix because $E_{3}$ is an upper triangular matrix with zero diagonal elements; while $J_{3}^{-1}$ and $J_{3}$ are both upper triangular matrices. The form of (28) is obtained by letting $F=E_{1}^{-1}J_{1}$.

Now consider system (2) with D=0, i.e.,

(32) $$ED^{\alpha }x\left(t\right)=Ax\left(t\right)+Bu\left(t\right),\;0<\alpha \leq 1,\;x\left(0\right)=v$$

(33) y(t)=Cx(t)

Let the system of (32) be regular. From Lemma 4, it follows that (32) can be rewritten as [37,38]:

(34) $$\left[\begin{array}{cc} I_{n_{1}} & 0\\ 0 & N \end{array}\right]\left[\begin{array}{c} D^{\alpha }w_{1}\left(t\right)\\ D^{\alpha }w_{2}\left(t\right) \end{array}\right]=\left[\begin{array}{cc} F & 0\\ 0 & I_{n_{2}} \end{array}\right]\left[\begin{array}{c} w_{1}\left(t\right)\\ w_{2}\left(t\right) \end{array}\right]+\left[\begin{array}{c} H\\ K \end{array}\right]u\left(t\right),$$

(35) $$w\left(t\right)=Q^{-1}x\left(t\right)\equiv {\left[\begin{array}{cc} w_{1}\left(t\right) & w_{2}\left(t\right) \end{array}\right]}^{T},$$

where $w_{1}\left(t\right)\in {\mathbb{R}}^{n_{1}}$; $w_{2}\left(t\right)\in {\mathbb{R}}^{n_{2}}$; and $PB={\left[\begin{array}{cc} H & K \end{array}\right]}^{T}$.

Thus, the following two subsystems are obtained:

(36) $$D^{\alpha }w_{1}\left(t\right)=Fw_{1}\left(t\right)+Hu\left(t\right),$$

(37) $$ND^{\alpha }w_{2}\left(t\right)=w_{2}\left(t\right)+Ku\left(t\right).$$

Since x(0)=v, therefore:

(38) $$w_{0}=Q^{-1}x\left(0\right)=Q^{-1}v\equiv {\left[v_{0}\;v_{1}\right]}^{T},$$

where $v_{0}\left(t\right)\in {\mathbb{R}}^{n_{1}}$ and $v_{1}\left(t\right)\in {\mathbb{R}}^{n_{2}}$.

The general solution of (36) is defined in terms of its fractional-order state equation (see (19)) as follows:

(39) $$w_{1}\left(t\right)=\phi_{\alpha 0}\left(t\right)v+\int_{0}^{t}\phi_{\alpha }\left(t-\tau \right)Hu\left(\tau \right)d\tau ,\;x\left(0\right)=v,$$

where

(40a) $$\phi_{\alpha 0}\left(t\right)=E_{\alpha ,1}\left(Ft^{\alpha }\right)=\sum_{k=0}^{\infty }\frac{{\left(Ft^{\alpha }\right)}^{k}}{\Gamma \left(k\alpha +1\right)},$$

(40b) $$\phi_{\alpha }\left(t\right)=t^{\alpha -1}E_{\alpha ,\alpha }\left(Ft^{\alpha }\right)=\sum_{k=0}^{\infty }\frac{F^{k}t^{\left(k+1\right)\alpha -1}}{\Gamma \left(\left(k+1\right)\alpha \right)}.$$

The solution of subsystem (37) is obtained using the property of the nilpotent matrix *N*. Thus, there are two cases to consider:

Case 1: N=0,

(41) $$\text{In this case},\;w_{2}\left(t\right)=-Ku\left(t\right).$$

Case 2: N≠0, 

To clarify this general case, let $N^{2}=0$. Pre-multiplying the second row of (34) by N yields:

(42) $$N^{2}D^{\alpha }w_{2}\left(t\right)=Nw_{2}\left(t\right)+NKu\left(t\right).$$

Now, differentiating both sides of (42) of order α (i.e., applying $D^{\alpha }$ to both sides) and using (35) yields:

(43) $$w_{2}\left(t\right)=-Ku\left(t\right)-NKD^{\alpha }u\left(t\right)+N^{2}D^{2\alpha }w_{2}\left(t\right).$$

Since $N^{2}=0$ by hypothesis, therefore:

(44) $$w_{2}\left(t\right)=-Ku\left(t\right)-NKD^{\alpha }u\left(t\right).$$

In general, since *N* is a nilpotent matrix, there exists an integer number *l* such that $N^{l}=0$. Now, pre-multiplying (34) by $N^{l-1}$ and using (35) yields:

(45) $$w_{2}\left(t\right)=-Ku\left(t\right)-\sum_{j=0}^{l-1}N^{j}KD^{j\alpha }u\left(t\right).$$

Substituting (40a) and (45) into (37) and (38) implies the following general solution of (34):

(46a) $$x\left(t\right)=Q\left[\begin{array}{c} I_{n_{1}}\\ 0_{n_{2}\times n_{1}} \end{array}\right](\phi_{\alpha 0}\left(t\right)v_{0}+\int_{0}^{t}\phi_{\alpha }\left(t-\tau \right)Hu\left(\tau \right)d\tau )+Q\left[\begin{array}{c} 0_{n_{1}\times n_{2}}\\ I_{n_{2}} \end{array}\right](-Ku\left(t\right)-\sum_{j=0}^{l-1}N^{j}KD^{j\alpha }u\left(t\right)),$$

where

(46b) $$\phi_{\alpha 0}\left(t\right)=E_{\alpha ,1}\left(Ft^{\alpha }\right)=\overset{\infty }{\underset{k=0}{\sum }}\frac{{\left(Ft^{\alpha }\right)}^{k}}{\Gamma \left(k\alpha +1\right)},$$

(46c) $$\phi_{\alpha }\left(t\right)=t^{\alpha -1}E_{\alpha ,\alpha }\left(At^{\alpha }\right)=\sum_{k=0}^{\infty }\frac{A^{k}t^{\left(k+1\right)\alpha -1}}{\Gamma \left(\left(k+1\right)\alpha \right)}.$$

Finally, the solution of y(t) follows directly from (46a) and (34).

## 5. Recursive form of FoLTI Systems with Regular Pencils

A recursive solution of FoLTI systems with regular pencils is first investigated by considering the unforced system (homogeneous) of (32) and by using proper Schur transformation matrices. The following theorem outlines the main results of this work.

**Theorem** **1.** *Consider a FoLTI homogeneous singular system with regular pencils of the following form:*

(47a) $$ED^{\alpha }x\left(t\right)=Ax\left(t\right),\;0<\alpha \leq 1,$$

(47b) y(t)=Cx(t),

*where*$E\in {\mathbb{R}}^{n\times n}$*is a singular matrix of rank*n−d<n; $x\left(t\right)\in {\mathbb{R}}^{n}$; $y\left(t\right)\in {\mathbb{R}}^{p}$; $A\in {\mathbb{R}}^{n\times n};\;C\in {\mathbb{R}}^{p\times n}$*with*rank C=p; *and with an initial condition*x(0)=v*. Let*$A_{\lambda }\equiv {\left(A-\lambda^{\alpha }E\right)}^{-1}A,\;E_{\lambda }\equiv {\left(A-\lambda^{\alpha }E\right)}^{-1}E$*; and let*$Q=\left[\begin{array}{cc} Q_{s} & \tilde{Q_{s}} \end{array}\right]\in {\mathbb{R}}^{n\times n}$*be a unitary matrix such that*$E_{\lambda }=QTQ^{*}=\left[\begin{array}{cc} Q_{s} & \tilde{Q_{s}} \end{array}\right]\left[\begin{array}{cc} G_{s} & D_{s}\\ 0 & N_{s} \end{array}\right]\left[\begin{array}{c} Q_{s}{}^{*}\\ {\tilde{Q_{s}}}^{*} \end{array}\right]$, *where*$G_{s}\in {\mathbb{R}}^{n-d\times n-d}$*is an invertible matrix and*$N_{s}\in {\mathbb{R}}^{d\times d}$*is a nilpotent matrix, which has all the zero eigenvalues of*$E_{\lambda }$*. Therefore, the general solution of (47) can be expressed as:*

(48) $$x\left(t\right)=Q_{s}E_{\alpha ,1}\left(\left(G_{s}{}^{-1}+\lambda^{\alpha }I\right)t^{\alpha }\right)Q_{s}{}^{*}\;v=Q_{s}\left[\overset{\infty }{\underset{k=0}{\sum }}\frac{{\left(\left(G_{s}{}^{-1}+\lambda^{\alpha }I\right)t^{\alpha }\right)}^{k}}{\Gamma \left(k\alpha +1\right)}\right]Q_{s}{}^{*}v$$

*and*

y(t)=Cx(t).

**Proof.**  The general solution of (47) can be obtained by transforming it into a recursive form using the Schur factorization structure. Since the pair (E,A) is assumed to be a regular pencil, from Lemma 1, there exists some λ∈ℂ such that $A-\lambda^{\alpha }E$ is invertible, which implies that $\det\left(A-\lambda^{\alpha }E\right)\neq 0$. Since $A_{\lambda }\equiv {\left(A-\lambda^{\alpha }E\right)}^{-1}A,$ and $E_{\lambda }\equiv {\left(A-\lambda^{\alpha }E\right)}^{-1}E$ by hypothesis, pre-multiplying (47a) by ${\left(A-\lambda^{\alpha }E\right)}^{-1}$ yields:

(49) $${\left(A-\lambda^{\alpha }E\right)}^{-1}ED^{\alpha }x\left(t\right)={\left(A-\lambda^{\alpha }E\right)}^{-1}A\;x\left(t\right).$$

System (47) can be rewritten as:

(50) $$E_{\lambda }D^{\alpha }x\left(t\right)=A_{\lambda }x\left(t\right).$$

Since

(51) $$A_{\lambda }={\left(A-\lambda^{\alpha }E\right)}^{-1}A={\left(A-\lambda^{\alpha }E\right)}^{-1}\left(A-\lambda^{\alpha }E+\lambda^{\alpha }E\right)=I+\lambda^{\alpha }E_{\lambda },$$

Then (50) is defined as:

(52) $$E_{\lambda }D^{\alpha }x\left(t\right)=\left(I+\lambda^{\alpha }E_{\lambda }\right)x\left(t\right).$$

Now, since $E_{\lambda }=QTQ^{*}=\left[\begin{array}{cc} Q_{s} & \tilde{Q_{s}} \end{array}\right]\left[\begin{array}{cc} G_{s} & D_{s}\\ 0 & N_{s} \end{array}\right]\left[\begin{array}{c} Q_{s}{}^{*}\\ {\tilde{Q_{s}}}^{*} \end{array}\right]$ by hypothesis, which is verified in [35], one may decompose the system states of (47a) to obtain:

(53) $$x\left(t\right)=Q_{s}x_{1}\left(t\right)+\tilde{Q_{s}}x_{2}\left(t\right),$$

where $x_{1}\in {\mathbb{R}}^{n-d},\;x_{2}\in {\mathbb{R}}^{d}$; *d* > 0.

Substituting (53) into (52) gives:

(54) $$E_{\lambda }\left[\begin{array}{cc} Q_{s} & \tilde{Q_{s}} \end{array}\right]D^{\alpha }\left[\begin{array}{c} x_{1}\left(t\right)\\ x_{2}\left(t\right) \end{array}\right]=\left(I+\lambda^{\alpha }E_{s}\right)\left[\begin{array}{cc} Q_{s} & \tilde{Q_{s}} \end{array}\right]\left[\begin{array}{c} x_{1}\left(t\right)\\ x_{2}\left(t\right) \end{array}\right].$$

Moreover, applying the Schur decomposition on (52) yields:

(55) $$\left[\begin{array}{cc} Q_{s} & \tilde{Q_{s}} \end{array}\right]\left[\begin{array}{cc} G_{s} & D_{s}\\ 0 & N_{s} \end{array}\right]\left[\begin{array}{c} Q_{s}{}^{*}\\ {\tilde{Q_{s}}}^{*} \end{array}\right]\left[\begin{array}{cc} Q_{s} & \tilde{Q_{s}} \end{array}\right]\left[\begin{array}{c} D^{\alpha }x_{1}\left(t\right)\\ D^{\alpha }x_{2}\left(t\right) \end{array}\right]=\left[\begin{array}{cc} Q_{s} & \tilde{Q_{s}} \end{array}\right]\left[\begin{array}{c} x_{1}\left(t\right)\\ x_{2}\left(t\right) \end{array}\right]+\left[\begin{array}{cc} Q_{s} & \tilde{Q_{s}} \end{array}\right]\left[\begin{array}{cc} \lambda^{\alpha }G_{s} & \lambda^{\alpha }D_{s}\\ 0 & \lambda^{\alpha }N_{s} \end{array}\right]\left[\begin{array}{c} Q_{s}{}^{*}\\ {\tilde{Q_{s}}}^{*} \end{array}\right]\left[\begin{array}{cc} Q_{s} & \tilde{Q_{s}} \end{array}\right]\left[\begin{array}{c} x_{1}\left(t\right)\\ x_{2}\left(t\right) \end{array}\right]$$

which leads to:

(56) $$\left[\begin{array}{cc} Q_{s} & \tilde{Q_{s}} \end{array}\right]\left[\begin{array}{cc} G_{s} & D_{s}\\ 0 & N_{s} \end{array}\right]\left[\begin{array}{c} D^{\alpha }x_{1}\left(t\right)\\ D^{\alpha }x_{2}\left(t\right) \end{array}\right]=\left[\begin{array}{cc} Q_{s} & \tilde{Q_{s}} \end{array}\right]\left[\begin{array}{c} x_{1}\left(t\right)\\ x_{2}\left(t\right) \end{array}\right]+\left[\begin{array}{cc} Q_{s} & \tilde{Q_{s}} \end{array}\right]\left[\begin{array}{cc} \lambda^{\alpha }G_{s} & \lambda^{\alpha }D_{s}\\ 0 & \lambda^{\alpha }N_{s} \end{array}\right]\left[\begin{array}{c} x_{1}\left(t\right)\\ x_{2}\left(t\right) \end{array}\right].$$

Now, since $Q=\left[\begin{array}{cc} Q_{s} & \tilde{Q_{s}} \end{array}\right]$ is invertible, we obtain:

(57) $$\left[\begin{array}{cc} G_{s} & D_{s}\\ 0 & N_{s} \end{array}\right]\left[\begin{array}{c} D^{\alpha }x_{1}\left(t\right)\\ D^{\alpha }x_{2}\left(t\right) \end{array}\right]=\left(I+\left[\begin{array}{cc} \lambda^{\alpha }G_{s} & \lambda^{\alpha }D_{s}\\ 0 & \lambda^{\alpha }N_{s} \end{array}\right]\right)\left[\begin{array}{c} x_{1}\left(t\right)\\ x_{2}\left(t\right) \end{array}\right]$$

Thus, (57) yields the following coupled equations:

(58) $$G_{s}D^{\alpha }x_{1}\left(t\right)=\left(1+\lambda^{\alpha }G_{s}\right)x_{1}\left(t\right)+\lambda^{\alpha }D_{s}x_{2}\left(t\right),$$

(59) $$N_{s}D^{\alpha }x_{2}\left(t\right)=\left(1+\lambda^{\alpha }N_{s}\right)x_{2}\left(t\right).$$

Since $G_{s}$ is invertible, (58) can be rewritten as:

(60) $$D^{\alpha }x_{1}\left(t\right)=\left(G_{s}{}^{-1}+\lambda^{\alpha }I\right)x_{1}\left(t\right)+\lambda^{\alpha }{G_{s}}^{-1}D_{s}x_{2}\left(t\right).$$

Moreover, since $N_{s}$ is a nilpotent matrix with $N_{s}{}^{d}=0$, pre-multiplying (59) by $N_{s}{}^{d-1}$ implies:

(61) $$0=N_{s}{}^{d}D^{\alpha }x_{2}\left(t\right)=\left(N_{s}{}^{d-1}+\lambda^{\alpha }N_{s}{}^{d}\right)x_{2}\left(t\right)=N_{s}{}^{d-1}x_{2}\left(t\right).$$

This implies that $N_{s}{}^{d-1}D^{\alpha }x_{2}\left(t\right)=0$. Again, we have:

(62) $$0=N_{s}{}^{d-1}D^{\alpha }x_{2}\left(t\right)=\left(N_{s}{}^{d-2}+\lambda^{\alpha }N_{s}{}^{d-1}\right)x_{2}\left(t\right)=N_{s}{}^{d-2}x_{2}\left(t\right).$$

This also implies that $N_{s}{}^{d-2}D^{\alpha }x_{2}\left(t\right)=0$. Repeating this process with decreasing powers of $N_{s}$ eventually leads to $x_{2}\left(t\right)=0$ for all t. Therefore, the subsystems (59) and (60), respectively, become:

(63) $$D^{\alpha }x_{1}\left(t\right)=\left(G_{s}{}^{-1}+\lambda^{\alpha }I\right)x_{1}\left(t\right)$$

and

(64) $$x_{2}\left(t\right)=0.$$

Obviously, according to the Schur basis of (53) and from (19–21), the recursive solution of (63) is given by:

(65) $$x\left(t\right)=Q_{s}E_{\alpha ,1}\left(\left(G_{s}{}^{-1}+\lambda^{\alpha }I\right)t^{\alpha }\right)Q_{s}{}^{*}\;v=Q_{s}\left[\overset{\infty }{\underset{k=0}{\sum }}\frac{{\left(\left(G_{s}{}^{-1}+\lambda^{\alpha }I\right)t^{\alpha }\right)}^{k}}{\Gamma \left(k\alpha +1\right)}\right]Q_{s}{}^{*}v$$

and

(66) y(t)=Cx(t).

Hence, the general solution (65) of the singular FoLTI systems with regular pencils is completely identified. □

## 6. Illustrative Examples

To illustrate the main ideas of this work, this section includes two numerical examples to highlight the main ideas of the proposed approach of solving singular FoLTI continuous systems.

**Example** **1.** *Consider the following singular FoLTI system, where* R *and* L *are constants, while* u(t) *is an input signal:*

(67a) $$LD^{\alpha }x_{3}\left(t\right)=u\left(t\right),$$

(67b) $$Rx_{2}\left(t\right)=u\left(t\right),$$

(67c) $$x_{1}\left(t\right)-x_{2}\left(t\right)-x_{3}\left(t\right)=0.$$

One may rewrite system (67) in the following form:

(68) $$\left[\begin{array}{ccc} 0 & 0 & L\\ 0 & 0 & 0\\ 0 & 0 & 0 \end{array}\right]D^{\alpha }\left[\begin{array}{c} x_{1}\left(t\right)\\ x_{2}\left(t\right)\\ x_{3}\left(t\right) \end{array}\right]=\left[\begin{array}{ccc} 0 & 0 & 0\\ 1 & -1 & -1\\ 0 & -R & 0 \end{array}\right]\left[\begin{array}{c} x_{1}\left(t\right)\\ x_{2}\left(t\right)\\ x_{3}\left(t\right) \end{array}\right]+\left[\begin{array}{c} 1\\ 0\\ 1 \end{array}\right]u\left(t\right),$$

where:

(69) $$E=\left[\begin{array}{ccc} 0 & 0 & L\\ 0 & 0 & 0\\ 0 & 0 & 0 \end{array}\right],\;A=\left[\begin{array}{ccc} 0 & 0 & 0\\ 1 & -1 & -1\\ 0 & -R & 0 \end{array}\right],\;\mathrm{and}\;B=\left[\begin{array}{c} 1\\ 0\\ 1 \end{array}\right].$$

Obviously, E is singular since det E=0. However, the pencil (E,A) is regular, because:

(70) $$\det\left(E\lambda^{\alpha }-A\right)=\left|\begin{array}{ccc} 0 & 0 & L\lambda^{\alpha }\\ -1 & 1 & 1\\ 0 & R & 0 \end{array}\right|=-RL\lambda^{\alpha }\neq 0.$$

From (46), the solution of system (68) is obtained as follows:

(71) $$\mathrm{Let}\;P=\left[\begin{array}{ccc} \frac{1}{L} & 0 & 0\\ 0 & 0 & \frac{-1}{R}\\ 0 & 1 & \frac{-1}{R} \end{array}\right],\;\mathrm{and}\;Q=\left[\begin{array}{ccc} 1 & 0 & 1\\ 0 & 1 & 0\\ 1 & 0 & 0 \end{array}\right];\;Q^{-1}=\left[\begin{array}{ccc} 1 & 0 & 1\\ 0 & 1 & 0\\ 1 & 0 & -1 \end{array}\right].$$

It follows that:

(72) $$PEQ=\left[\begin{array}{ccc} 1 & 0 & 0\\ 0 & 0 & 0\\ 0 & 0 & 0 \end{array}\right],\;PAQ=\left[\begin{array}{ccc} 0 & 0 & 0\\ 0 & 1 & 0\\ 0 & 0 & 1 \end{array}\right],\;PB=\left[\begin{array}{c} \frac{1}{L}\\ \frac{-1}{R}\\ \frac{-1}{R} \end{array}\right].$$

From (28), system (69) can be transformed to the following form:

(73) $$\left[\begin{array}{ccc} 1 & 0 & 0\\ 0 & 0 & 0\\ 0 & 0 & 0 \end{array}\right]\left[\begin{array}{ccc} 1 & 0 & 1\\ 0 & 1 & 0\\ 1 & 0 & -1 \end{array}\right]D^{\alpha }\left[\begin{array}{c} x_{1}\left(t\right)\\ x_{2}\left(t\right)\\ x_{3}\left(t\right) \end{array}\right]=\left[\begin{array}{ccc} 0 & 0 & 0\\ 0 & 1 & 0\\ 0 & 0 & 1 \end{array}\right]\left[\begin{array}{ccc} 1 & 0 & 1\\ 0 & 1 & 0\\ 1 & 0 & -1 \end{array}\right]\left[\begin{array}{c} x_{1}\left(t\right)\\ x_{2}\left(t\right)\\ x_{3}\left(t\right) \end{array}\right]+\left[\begin{array}{c} \frac{1}{L}\\ \frac{-1}{R}\\ \frac{-1}{R} \end{array}\right]u\left(t\right).$$

In light of (34) and (35), further transformation of (73), respectively, yields:

(74) $$\left[\begin{array}{ccc} 1 & 0 & 0\\ 0 & 0 & 0\\ 0 & 0 & 0 \end{array}\right]D^{\alpha }\left[\begin{array}{c} w_{1}\left(t\right)\\ w_{2}\left(t\right) \end{array}\right]=\left[\begin{array}{ccc} 0 & 0 & 0\\ 0 & 1 & 0\\ 0 & 0 & 1 \end{array}\right]\left[\begin{array}{c} w_{1}\left(t\right)\\ w_{2}\left(t\right) \end{array}\right]+\left[\begin{array}{c} H\\ K \end{array}\right]u\left(t\right)$$

with

(75) $$w\left(t\right)=\left[\begin{array}{ccc} 1 & 0 & 1\\ 0 & 1 & 0\\ 1 & 0 & -1 \end{array}\right]\left[\begin{array}{c} x_{1}\left(t\right)\\ x_{2}\left(t\right)\\ x_{3}\left(t\right) \end{array}\right],\;\left[\begin{array}{c} H\\ K \end{array}\right]=\left[\begin{array}{c} \frac{1}{L}\\ \frac{-1}{R}\\ \frac{-1}{R} \end{array}\right].$$

Thus:

(76a) $$\begin{array}{l} \begin{array}{c} D^{\alpha }w_{1}\left(t\right)=D^{\alpha }x_{3}\left(t\right)=\frac{1}{L}u\left(t\right) \end{array} \end{array},$$

(76b) $$w_{2}\left(t\right)=\left[\begin{array}{c} x_{2}\left(t\right)\\ x_{1}\left(t\right)-x_{3}\left(t\right) \end{array}\right]=-\left[\begin{array}{c} \frac{-1}{R}\\ \frac{-1}{R} \end{array}\right]u\left(t\right).$$

Using (40), the general solution of (67) is given by:

(77) $$\left[\begin{array}{c} x_{1}\left(t\right)\\ x_{2}\left(t\right)\\ x_{3}\left(t\right) \end{array}\right]=\left[\begin{array}{l} \frac{1}{R}u\left(t\right)+x_{3}\left(0\right)+\frac{1}{L\Gamma \left(\alpha \right)}\int_{0}^{t}{\left(t-\tau \right)}^{\alpha -1}u\left(\tau \right)d\tau \\ \frac{1}{R}u\left(t\right)\\ x_{3}\left(0\right)+\frac{1}{L\Gamma \left(\alpha \right)}\int_{0}^{t}{\left(t-\tau \right)}^{\alpha -1}u\left(\tau \right)d \end{array}\right]$$

**Example** **2.** *Consider system (47) for* 0<α≤1 *where:*

(78) $$E=[\begin{array}{cccc} 1 & 0 & 0 & 0\\ 0 & 0 & 1 & 0\\ 0 & 0 & 0 & 0\\ 0 & 0 & 0 & 0 \end{array}],\;A=[\begin{array}{cccc} 0 & 0 & 0 & 0\\ 1 & 0 & 0 & 0\\ 0 & 1 & 0 & 0\\ 0 & 0 & 0 & 1 \end{array}],\;C=\left[1\;\;0\;\;1\;\;0\right],$$

*subject to the initial condition*

(79) $$x\left(0\right)=v=\left[\begin{array}{c} 1\\ 1\\ 0\\ 1 \end{array}\right].$$

Notice that E is a singular matrix since det E=0, and the pencil (E,A) is regular, because:

(80) $$\det\left(A-\lambda^{\alpha }E\right)=\left|\begin{array}{cccc} -\lambda^{\alpha } & 0 & 0 & 0\\ 1 & 0 & -\lambda^{\alpha } & 0\\ 0 & 1 & 0 & 0\\ 0 & 0 & 0 & 1 \end{array}\right|=-\lambda^{2\alpha }\neq 0.$$

Observe that (78) represents the parameters of a singular FoLTI regular system. If follows from (52) that:

(81) $$E_{s}={\left(A-\lambda^{\alpha }E\right)}^{-1}E=[\begin{array}{cccc} -\frac{1}{\lambda^{\alpha }} & 0 & 0 & 0\\ 0 & 0 & 1 & 0\\ -\frac{1}{\lambda^{2\alpha }} & 0 & -\frac{1}{\lambda^{\alpha }} & 0\\ 0 & 0 & 0 & 0 \end{array}].$$

Thus, the Schur factorization for $E_{s}$ is:

(82) $$Q=[\begin{array}{cccc} 0 & 1 & 0 & 0\\ 0 & 0 & 1 & 0\\ 1 & 0 & 0 & 0\\ 0 & 0 & 0 & 1 \end{array}],\;T=\left[\begin{array}{cccc} \frac{-1}{\lambda^{\alpha }} & \frac{-1}{\lambda^{2\alpha }} & 0 & 0\\ 0 & \frac{-1}{\lambda^{\alpha }} & 0 & 0\\ 0 & 0 & 0 & 0\\ 0 & 0 & 0 & 0 \end{array}\right],\;Q^{*}=[\begin{array}{cccc} 0 & 0 & 1 & 0\\ 1 & 0 & 0 & 0\\ 0 & 1 & 0 & 0\\ 0 & 0 & 0 & 1 \end{array}].$$

That is:

(83) $$Q_{s}=[\begin{array}{cc} 0 & 1\\ 0 & 0\\ 1 & 0\\ 0 & 0 \end{array}],\;\tilde{Q_{s}}=[\begin{array}{cc} 0 & 0\\ 1 & 0\\ 0 & 0\\ 0 & 1 \end{array}],\;G_{s}=\left[\begin{array}{cc} \frac{-1}{\lambda^{\alpha }} & \frac{-1}{\lambda^{2\alpha }}\\ 0 & \frac{-1}{\lambda^{\alpha }} \end{array}\right],\;D_{s}=N_{s}=\left[\begin{array}{cc} 0 & 0\\ 0 & 0 \end{array}\right].$$

Moreover:

(84) $$\left(G_{s}{}^{-1}+\lambda^{\alpha }I\right)t^{\alpha }=\left[\begin{array}{cc} 0 & t^{\alpha }\\ 0 & 0 \end{array}\right].$$

Using (65), the general solution is given by:

(85) $$x\left(t\right)=Q_{s}E_{\alpha ,1}\left(\left(G_{s}{}^{-1}+\lambda^{\alpha }I\right)t^{\alpha }\right)Q_{s}{}^{*}\;v=[\begin{array}{cc} 0 & 1\\ 0 & 0\\ 1 & 0\\ 0 & 0 \end{array}]\left[\sum_{k=0}^{\infty }\frac{{\left[\begin{array}{cc} 0 & t^{\alpha }\\ 0 & 0 \end{array}\right]}^{k}}{\Gamma \left(k\alpha +1\right)}\right]\left[\begin{array}{cccc} 0 & 0 & 1 & 0\\ 1 & 0 & 0 & 0 \end{array}\right][\begin{array}{c} 1\\ 1\\ 0\\ 1 \end{array}].$$

Now, since:

(86) $${\left[\begin{array}{cc} 0 & t^{\alpha }\\ 0 & 0 \end{array}\right]}^{k}=0,\;k=2,\;3,\;4,\;\ldots$$

Thus, (85) reduces to:

(87) $$x\left(t\right)={\left[\begin{array}{cccc} 0 & 0 & \frac{t^{\alpha }}{\Gamma \left(\alpha +1\right)} & 0 \end{array}\right]}^{T}$$

Finally, from (77) and (87), $y\left(t\right)=Cx\left(t\right)=\;\frac{t^{\alpha }}{\Gamma \left(\alpha +1\right)}$.

## 7. Conclusions

The general solution of FoLTI continuous systems is introduced in the sense of the Caputo definition of fractional order derivative using the Adomian Decomposition Method (ADM). The same approach is extended to obtain the general solution of singular FoLTI continuous systems with regular pencils. This approach benefits from the structure of the canonical form of the system state matrices. Using the Schur decomposition, the system matrices were transformed to separate the static variables from the dynamic variables. Hence, a recursive technique is implemented to uniquely define the general solutions of both the dynamic and the static parts of the system. The case of singular FoLTI systems with singular pencils is left for further development.
